# Electroclinical and Multimodality Neuroimaging Characteristics and Predictors of Post-Surgical Outcome in Focal Cortical Dysplasia Type IIIa

**DOI:** 10.3389/fbioe.2021.810897

**Published:** 2022-01-10

**Authors:** Lingling Zhang, Hailing Zhou, Wei Zhang, Xueying Ling, Chunyuan Zeng, Yongjin Tang, Jiefeng Gan, Qinghua Tan, Xiangshu Hu, Hainan Li, Baijie Cheng, Hao Xu, Qiang Guo

**Affiliations:** ^1^ Department of Nuclear Medicine, PET/CT-MRI Center, Center of Cyclotron and PET Radiopharmaceuticals, The First Affiliated Hospital of Jinan University, Guangzhou, China; ^2^ Department of Neurology, Affiliated Hangzhou First People’s Hospital, Zhejiang University School of Medicine, Zhejiang, China; ^3^ Epilepsy Center, Guangdong 999 Brain Hospital, Guangzhou, China; ^4^ Department of Pathology, Guangdong 999 Brain Hospital, Guangzhou, China

**Keywords:** characteristics, predictors, postoperative outcome, focal cortical dysplasia type IIIa, drug-resistant epilepsy

## Abstract

Focal cortical dysplasia (FCD) type IIIa is an easily ignored cause of intractable temporal lobe epilepsy. This study aimed to analyze the clinical, electrophysiological, and imaging characteristics in FCD type IIIa and to search for predictors associated with postoperative outcome in order to identify potential candidates for epilepsy surgery. We performed a retrospective review including sixty-six patients with FCD type IIIa who underwent resection for drug-resistant epilepsy. We evaluated the clinical, electrophysiological, and neuroimaging features for potential association with seizure outcome. Univariate and multivariate analyses were conducted to explore their predictive role on the seizure outcome. We demonstrated that thirty-nine (59.1%) patients had seizure freedom outcomes (Engel class Ia) with a median postsurgical follow-up lasting 29.5 months. By univariate analysis, duration of epilepsy (less than 12 years) (*p* = 0.044), absence of contralateral insular lobe hypometabolism on PET/MRI (*p*_Log-rank_ = 0.025), and complete resection of epileptogenic area (*p*_Log-rank_ = 0.004) were associated with seizure outcome. The incomplete resection of the epileptogenic area (hazard ratio = 2.977, 95% CI 1.218–7.277, *p* = 0.017) was the only independent predictor for seizure recurrence after surgery by multivariate analysis. The results of past history, semiology, electrophysiological, and MRI were not associated with seizure outcomes. Carefully included patients with FCD type IIIa through a comprehensive evaluation of their clinical, electrophysiological, and neuroimaging characteristics can be good candidates for resection. Several preoperative factors appear to be predictive of the postoperative outcome and may help in optimizing the selection of ideal candidates to benefit from epilepsy surgery.

## Introduction

Epilepsy is one of the most common severe chronic neurological disorders and is considered a severe public health concern (Blumcke et al., 2017). Drug-resistant epilepsy accounts for at least one-third of people with epilepsy (Montaz-Rosset et al., 2019). The most familiar developmental cortical malformation that leads to intractable epilepsy is focal cortical dysplasia (FCD) (Palmini and Holthausen, 2013). FCD is the most common histopathology diagnosis in children and the third most familiar etiology in adult patients undergoing epilepsy resection (Hauptman and Mathern, 2012). With the development of neuroimaging techniques, especially MRI and ^18^F-FDG PET (Tassi et al., 2002), more studies have shown that a higher incidence of FCD than previously diagnosed have enhanced the preoperative recognition and classification of these malformations (Widdess-Walsh et al., 2006). These patients have a serious seizure burden: more than 60% suffer from daily seizures, and a large number of FCD patients have onset of epilepsy by the age of 16 years (Chapman et al., 2005).

For these refractory epilepsy patients associated with FCD, the resection of epilepsy has become the most promising alternative therapeutic option to achieve seizure control in recent decades. Among the FCD patients with resection, postoperative seizure control ranged from 52% to 67% (D. W. Kim et al., 2009; Lerner et al., 2009). Incomplete resection of the epileptogenic area could lead to persistent seizures (Najm et al., 2013). These epilepsy individuals are confronted with difficulties because operative seizure outcomes cannot be predicted accurately before operation. Therefore, in patients with intractable epilepsy and FCD, the combination of clinical, electrophysiological data and multimodality neuroimaging features during a detailed preoperative assessment may help to precisely localize the epileptogenic zone (EZ) (Pittau et al., 2014) and predict seizure outcome after surgery (Mintzer and Sperling, 2008).

However, previous researches have focused on investigating the predictors of postoperative outcomes for isolated FCD patients or dual pathology using Palmini’s FCD classification (Chang et al., 2011); thus, the predictors of postoperative seizure outcome in FCD associated with other principal lesions according to new classification (Blumcke et al., 2011), especially newly classified FCD type IIIa [FCD type I coexisting with hippocampal sclerosis (HS) in the temporal lobe, which is also a common pathology and different from isolated FCD in temporal lobe epilepsy (TLE) (Marusic et al., 2007)], are less well discussed (Fauser et al., 2015). Although it is very critical to explore the predictors in identifying perfect candidates for epilepsy surgery and evaluating the postoperative outcome of individual patients, there have been only restricted data about the predictive factors involved in the surgical therapy of intractable FCD type IIIa.

In our study, the demographic, clinical, and electrophysiological characteristics, the neuroimaging features, and the postoperative outcome were reviewed in the patients with histologically confirmed FCD type IIIa in our epilepsy center. The goal of the current study was to determine the reliable predictive factors for seizure freedom outcome using the Kaplan–Meier (KM) survival analysis and multivariate Cox proportional hazards model in order to better recognize potential candidates for epilepsy surgery.

## Materials and Methods

### Study Subjects

Our study retrospectively analyzed consecutive individuals diagnosed with medically intractable TLE and FCD type IIIa as determined by histopathology who underwent resective epilepsy surgery at our epilepsy center from January 1, 2014, until April 31, 2019. Drug-resistant epilepsy was diagnosed according to the criteria proposed by International League Against Epilepsy (ILAE), which fulfilled the definition “failure of adequate trials of two tolerated and appropriately chosen anti-epileptic drugs (AEDs) schedules (whether as monotherapies or in combination) to achieve seizure freedom” (Kwan et al., 2010).

The inclusion criteria were as follows: 1) all FCD type IIIa (HS-related FCD type I in the temporal lobe) was diagnosed by histopathology results after surgery according to the 2011 ILAE classification system for FCD (Blumcke et al., 2011); 2) postoperative follow-up was at least 12 months after surgery; and 3) either adult or children patients were included.

Exclusion criteria were as follows: 1) isolated FCD type I and II; 2) FCD in other brain lobes or multiple brain lobes; 3) hemispheric dysplasia, tuberous sclerosis, and periventricular nodular heterotopias; 4) isolated HS; 5) HS associated with tumor, vascular malformation or other early lesions (e.g., trauma, ischemic injury, and encephalitis) in the temporal lobe.

### Video Electroencephalogram and Stereoelectroencephalography

All patients underwent long-term scalp video electroencephalogram (VEEG) recording with 21 channels based on the international 10–20 system, with attached anterior temporal electrodes. Interictal and ictal scalp VEEG was recorded in each patient, and at least two habitual seizures during monitoring were reviewed for all patients. Ictal rhythm/interictal spike in the electrodes of an epileptogenic lobe or two adjacent electrodes was considered as a localizing pattern of ictal-onset rhythm/interictal spike.

Stereoelectroencephalography (SEEG) monitoring was recommended if the presurgical noninvasive evaluation was disconcordant, there was no visible causative lesion seen on MRI, or eloquent cortex was included. Some patients were implanted with electrodes in areas that could be associated with the EZ using robotic stereotactic assistance (ROSA) system. Long-term recordings were conducted after implanting electrodes utilizing VEEG monitoring system. And the EZ was defined as the regions covered by the electrodes that presented ictal discharge patterns that occurred either before or simultaneously with clinical behavior (Seo et al., 2011).

### Imaging

All epilepsy patients underwent sequential PET/MRI, conducted with CT-based attenuation correction and MRI using a trimodality PET/CT-MRI system (full ring, time-of-flight discovery PET/CT 690, 3T Discovery MR 750, GE Healthcare, Chicago, IL, USA) in the PET/CT-MRI Center at The First Affiliated Hospital of Jinan University. MRI was conducted first on a 3.0-T MR scanner (GE Discovery 750, Milwaukee, WI, USA). The interictal PET/CT scans for all patients were conducted using a GE Discovery PET-CT 690 system. All patients had clinical seizures no less than 24 h before the PET scan to exclude possible effects on PET sensitivity, and VEEG monitoring was not performed simultaneously with the PET scan. ^18^F-FDG PET visual analysis was semiquantitative and performed by two experienced nuclear physicians on the GE AW 4.6 workstation during the preoperation evaluation and before invasive SEEG. ^18^F-FDG PET and MRI data were sent to a dedicated review workstation (GE Advantage workstation, version 4.6; GE Healthcare Life Sciences GE), also evaluated by two experienced nuclear physicians.

### Surgery and Pathology

All patients underwent resection after the review of the presurgical evaluation information in a multidisciplinary case discussion. The resection range included the onset zone monitored by intracranial electrodes, the focal dysplastic area adjacent to the zone, and the hypometabolism region around the onset zone. Generally speaking, if the epileptogenic lesion is located in the mesial temporal lobe and temporal neocortex, the involved neocortex and mesial structures needed to be removed. We usually perform standardized anterior temporal lobe resection (ATLR) in our center. The temporal neocortex (4–5 cm on the dominant temporal cortex and 5–6 cm on the nondominant temporal cortex), in combination with the mesial temporal structures (consisting of the hippocampus, parahippocampal gyrus, and amygdala), was removed (Kemerdere et al., 2018). Some patients underwent extended temporal lobectomy and amygdalo-hippocampectomy on the basis of the results of SEEG. All patients underwent MRI to evaluate the completeness of resection after surgery. Complete excision was defined by resection of all of the MRI lesions and seizure onset zones defined by SEEG according to postoperative MRI. Moreover, almost all of these patients had changes in the medial structure, such as HS, which required complete resection. If postoperative MRI showed residual medial structure, it was considered incomplete resection.

The histopathological diagnosis was acquired by disposing of the surgical samples, as previously described (Chang et al., 2011). Histopathology of FCD was classified using ILAE 2011 classification guideline (Blumcke et al., 2011), as follows: FCD type IIIa refers to the temporal cortical dyslamination in combination with HS.

### Surgical Outcomes

Seizure outcome for all patients after surgery was determined by a review of clinic visits or telephone contacts. All individuals were followed up until April 31, 2020. Seizure outcome classifications after operation were made using Engel’s classification (Engel, 1993): Engel class Ia, completely seizure free after surgery; and Engel class I, seizure free or auras only or convulsions with drug withdrawal only. Patients were considered seizure free only if they never experienced seizures or auras throughout the follow-up period (Engel class Ia outcome), and acute postoperative seizures within the first week were excluded (Cahill et al., 2019).

### Statistical Analyses

The study population was divided into two groups including seizure freedom and seizure recurrence based on the prognosis at the last follow-up. For exploratory purposes, the continuous variables were compared between these two groups using the nonparametric Mann–Whitney U test due to non-normal distributions. Categorical variables were compared between two groups using a chi-square test or Fisher’s exact test, as appropriate.

KM survival curves were used to characterize the probability of remaining seizure free after surgery over time. First, KM survival analysis was performed to investigate the probability of seizure-free survival in the overall group. All cases lost to follow-up were censored at the date of last clinical contact. Then, the differences between groups in seizure freedom distributions were compared using the log-rank test for the univariate analysis. Variables with *p* < 0.1 in univariate analysis were then included in the multivariate Cox proportional regression model to analyze independent prognostic effect for seizure recurrence, Mantel–Haenszel hazard ratio (HR), and 95% CI were calculated.

All data analyses were conducted using the IBM SPSS 19.0 software package (SPSS Inc., Chicago, IL, USA) and GraphPad Prism, version 8.0 (GraphPad Software, La Jolla, CA, USA). A two-tailed *p* < 0.05 was considered statistically significant for all statistical tests.

## Results

### Patient Demographic and Clinical Characteristics

**FIGURE 1:**
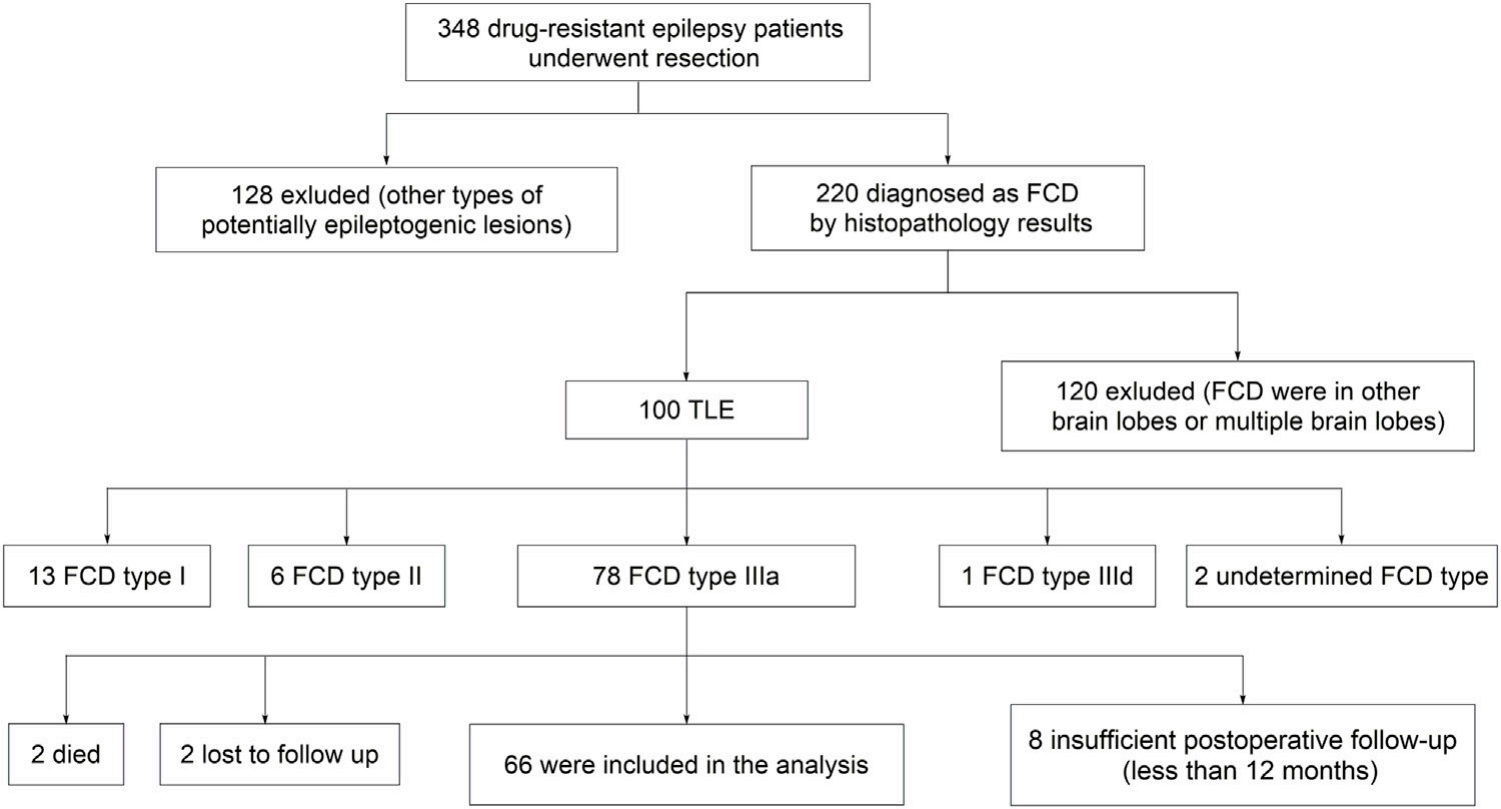
Flowchart of patient selection. FCD, focal cortical dysplasia; TLE, temporal lobe epilepsy.

We first selected three hundred and forty-eight drug-resistant epilepsy patients who underwent resection in our epilepsy center. Then we excluded patients with other types of potentially epileptogenic lesions (*n* = 128), FCD in other brain lobes or multiple brain lobes (*n* = 120), other types of FCD in temporal lobe (*n* = 22), insufficient postoperative follow-up (*n* = 8), undetermined death (*n* = 2), and lost to follow-up (*n* = 2). Finally, sixty-six patients met the inclusion criteria (Figure 1). The patients’ basic demographics and clinical characteristics are summarized in Table 1.

**TABLE 1:** **|** Demographic and clinical characteristics.

Variable	Outcome		*p*-Value
—	Seizure freedom	Seizure recurrence	—
No. of patients	39 (59.1%)	27 (40.9%)	—
Gender	—	—	0.756^a^
Male	26 (66.7%)	17 (63.0%)	—
Female	13 (33.3%)	10 (37.0%)	—
Duration of follow-up (months), median (IQR)	29.0 (22.0–40.5)	33.0 (22.0–42.0)	0.953^b^
Age at operation (years), median (IQR)	24.0 (16.0–29.0)	23.0 (19.0–28.0)	0.629^b^
Age at seizure onset (years), median (IQR)	12.0 (4.0–18.0)	9.0 (5.0–14.0)	0.368^b^
Duration of epilepsy (years), median (IQR)	10.0 (6.0–15.0)	12.0 (9.0–20.0)	0.098^b^
Duration of epilepsy (＜12 years)	27 (69.2%)	12 (44.4%)	0.044^a^ ^*^
Presence of FC	6 (15.4%)	7 (25.9%)	0.290^a^
Early brain injury	0 (0%)	0 (0%)	—
Family history	2 (5.1%)	0 (0%)	0.509^c^
Cerebral anoxia	0 (0%)	0 (0%)	—
CNS infection	4 (10.3%)	3 (11.1%)	1.000^a^
Head trauma	11 (28.2%)	8 (29.6%)	0.900^a^
Seizure type			
FIAS	26 (66.7%)	15 (55.6%)	0.360^a^
FAS	1 (2.6%)	0 (0%)	1.000^c^
FBTCS	12 (30.8%)	12 (44.4%)	0.256^a^
Auras	23 (59.0%)	17 (63.0%)	0.744^a^
Fear	6 (15.4%)	1 (3.7%)	0.268^a^
Epigastric	4 (10.3%)	4 (14.8%)	0.862^a^
Semiology			
Hypermotor	9 (23.1%)	8 (29.6%)	0.549^a^
Autonomic	23 (59.0%)	19 (70.4%)	0.344^a^
BATS	3 (7.7%)	0 (0%)	0.382^a^
Unilateral deviation	8 (20.5%)	6 (22.2%)	0.867^a^
Automatisms	28 (71.8%)	16 (59.3%)	0.288^a^
Seizure frequency			
Daily	12 (30.8%)	6 (22.2%)	0.443^a^
Weekly	8 (20.5%)	5 (18.5%)	0.841^a^
Monthly	19 (48.7%)	16 (59.3%)	0.399^a^
Type of epilepsy surgery (ATLR)	16 (41.0%)	8 (29.6%)	0.344^a^
Left side of surgery	26 (66.7%)	15 (55.6%)	0.360^a^
Complete resection of epileptogenic area	37 (94.9%)	20 (74.1%)	0.040^a^ ^*^

Note. Categorical variables are n (%) unless stated otherwise.

IQR, interquartile range; FC, febrile convulsion; CNS, central nervous system; FIAS, focal impaired awareness seizure; FAS, focal aware seizure; FBTCS, focal to bilateral tonic–clonic seizure; BATS, bilateral asymmetric rigidity; ATLR, anterior temporal lobe resection.

*Statistically significant.

a Chi-square test.

b Mann–Whitney U test.

c Fisher’s exact test.

The most common preoperative seizure type based on the new classification method of seizure types (Fisher et al., 2017) was focal impaired awareness seizure (FIAS) (62.1%), followed by focal to bilateral tonic–clonic seizure (FBTCS) (36.4%). The frequency of seizures before surgery was divided into three categories: daily seizures (≥1 seizure each day), weekly seizures (≥1 seizure each week), and monthly seizures (≥1 seizure each month) (Chang et al., 2011). Among these, eighteen (27.3%) patients had daily seizures. Finally, twenty-four patients (36.4%) underwent ATLR, and fifty-seven (86.4%) patients underwent complete resection of epileptogenic area.

### Preoperative Electrophysiological and Neuroimaging Characteristics

The electrophysiological characteristics of these patients are demonstrated in Table 2. Focal interictal epileptiform discharges (IEDs) on scalp VEEG were localized in thirty-two (48.5%) patients. Moreover, localized ictal onset zone on scalp EEG was clearly localized in twenty-seven (40.9%) patients. SEEG was conducted in sixty (90.9%) cases using the ROSA system (Medtech, Montpellier, France), confirming that ictal discharge patterns occurred confined to either the mesial temporal lobe or neocortical areas.

**TABLE 2:** **|** Electrophysiological and multimodality neuroimaging characteristics.

Variable	Outcome		*p*-Value
—	Seizure freedom	Seizure recurrence	—
Interictal scalp VEEG, focal IED	18 (46.2%)	14 (51.9%)	0.649^a^
Bilateral temporal lobe IED	6 (15.4%)	6 (22.2%)	0.701^a^
Ictal scalp VEEG, localized ictal onset zone	14 (35.9%)	13 (48.1%)	0.320^a^
Invasive SEEG (number)	37 (94.9%)	23 (85.2%)	0.363^a^
MRI			
Gray–white blurring	5 (12.8%)	1 (3.7%)	0.406^a^
Cortical thickening	1 (2.6%)	2 (7.4%)	0.743^a^
Hippocampal sclerosis	26 (66.7%)	16 (59.3%)	0.539^a^
Subcortical T2/FLAIR abnormality	2 (5.1%)	0 (0%)	0.509^b^
MRI-Ictal VEEG concordance	15 (38.5%)	8 (29.6%)	0.459^a^
PET/MRI coregistration			
Presence of contralateral TLH	3 (7.7%)	2 (7.4%)	1.000^a^
Contralateral frontal lobe hypometabolism	3 (7.7%)	5 (18.5%)	0.346^a^
Bilateral frontal lobe hypometabolism	2 (5.1%)	4 (14.8%)	0.363^a^
Contralateral insular lobe hypometabolism	0 (0%)	3 (11.1%)	0.126^a^

Note. Categorical variables are n (%) unless stated otherwise.

VEEG, video electroencephalogram; IED, interictal epileptiform discharges; FLAIR, fluid-attenuated inversion recovery; TLH, temporal lobe hypometabolism.

a Chi-square test.

b Fisher’s exact test.

We also assessed the preoperative neuroimaging features in overall cases (Table 2). Structural MRI could only detect thirteen (19.7%) patients with FCD type IIIa. Approximately 63.6% of patients had typical HS lesions on structural MRI, whereas only six (9.1%) patients presented gray–white matter transition blurring. Moreover, cortical thickening and subcortical T2/fluid-attenuated inversion recovery (FLAIR) abnormality were significantly less frequent in only three (4.5%) and two (3%) patients, respectively. MRI findings were closely concordant with ictal scalp VEEG onset pattern in twenty-three (34.8%) patients.

Moreover, we further analyzed the metabolism pattern in these sixty-six patients using ^18^F-FDG PET/MRI coregistration. Contralateral temporal lobe hypometabolism (TLH) was observed in only five (7.6%) patients. Contralateral frontal lobe hypometabolism and bilateral frontal lobe hypometabolism were also found in about eight (12.1%) and six (9.1%) patients, respectively. We also demonstrated that contralateral insular lobe hypometabolism was relatively infrequent in three (4.5%) patients.

### Postoperative Seizure Outcomes

**FIGURE 2:**
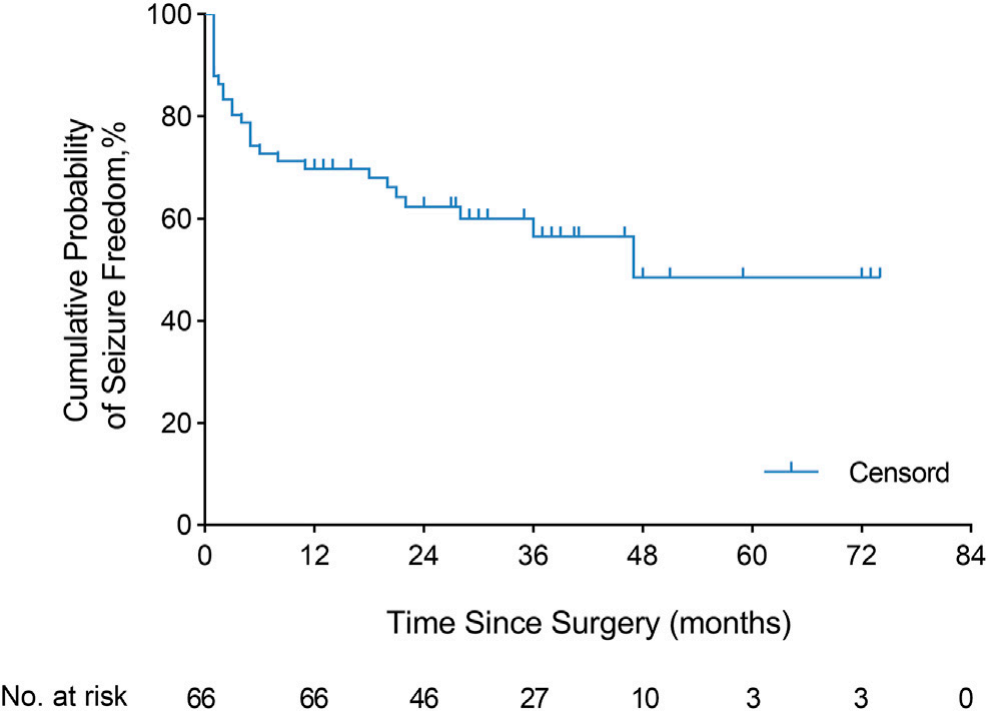
Engel class Ia seizure freedom. Kaplan–Meier curve demonstrated the seizure freedom outcome after resection in the overall population (66 patients). The cumulative probability of continuous seizure freedom was 69.7% (95% CI, 58.6–80.8%), 62.3% (95% CI, 50.3–74.4%), 56.5% (95% CI, 43.0–70.0%), and 48.4% (95% CI, 29.8–67.1%) at 12, 24, 36, and 60 months or more after surgery, respectively.

The continuous Engel class Ia seizure freedom outcome after the operation is illustrated using KM curves (Figure 2). The cumulative probability of seizure freedom survival was 69.7% (95% CI, 58.6–80.8%), 62.3% (95% CI, 50.3–74.4%), 56.5% (95% CI, 43.0–70.0%), and 48.4% (95% CI, 29.8–67.1%) at 12, 24, 36, and 60 months or more after surgery, respectively.

**FIGURE 3:**
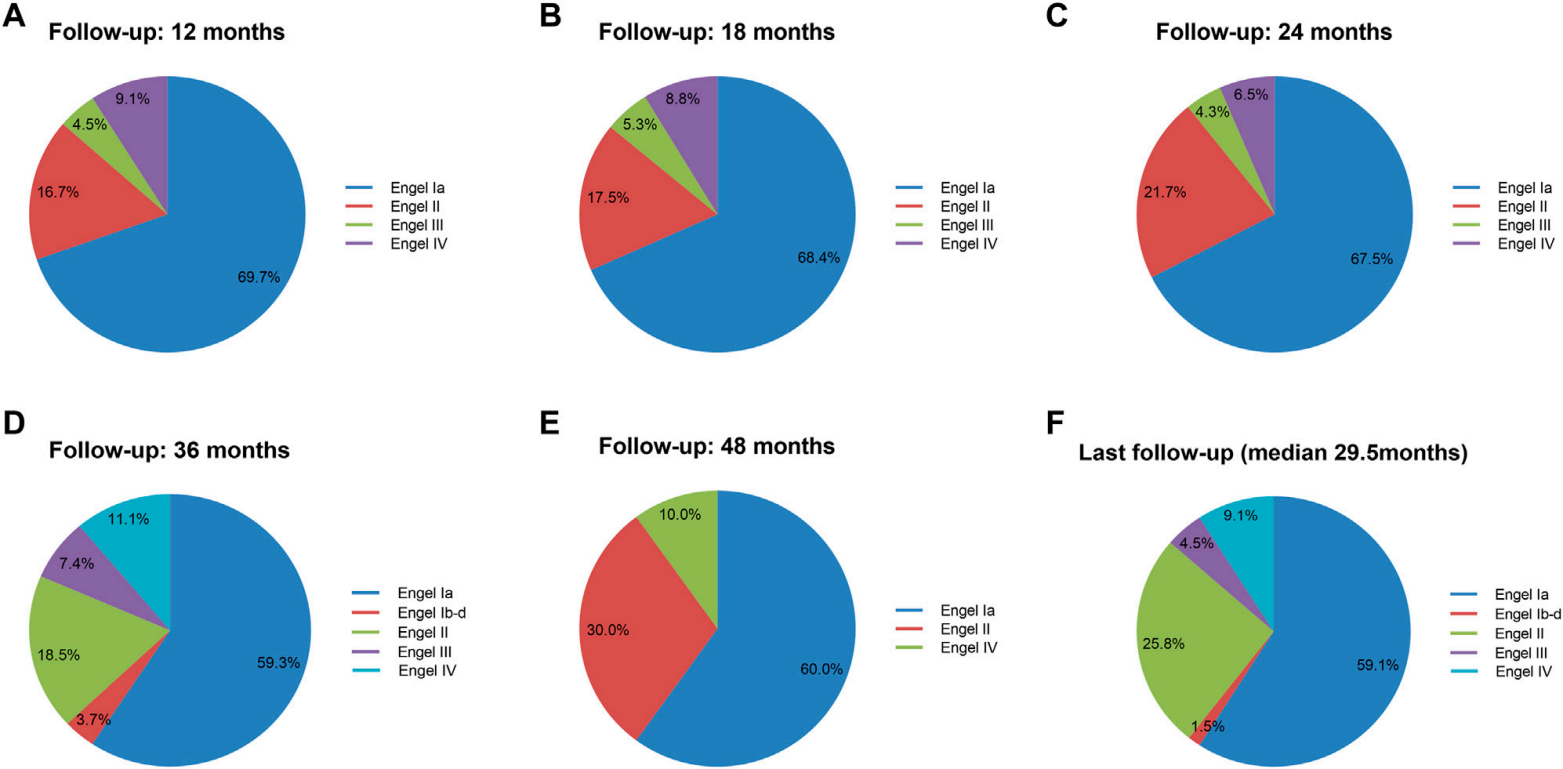
Engel classification outcome during postoperative follow-up. Engel classification outcomes at postoperative 12, 18, 24, 36, and 48 months and last follow-up. Total numbers of investigated cohorts and the rates of cohorts were illustrated for each Engel class outcome. The Engel class Ia outcome worsened with time.

To evaluate in detail the seizure outcomes after surgery, we further investigated each Engel classification outcome distribution at different follow-up times (≥12 months) in sixty-six patients (Figure 3). At the last follow-up time (median, 29.5 months), thirty-nine patients (59.1%) were free of seizures and auras (Engel class Ia) after surgery, and one patient (1.5%) had Engel class Ib–d outcome. Seventeen patients (25.8%) had Engel class II outcome, three patients (4.5%) had Engel class III outcome, and six patients (9.1%) had an Engel class IV outcome.

### Predictors of Seizure Freedom Outcome

We firstly performed a univariate analysis of the predictive factors influencing the seizure outcome (Table 1, 2), and the postoperative seizure outcome was classified as two groups including seizure freedom and seizure recurrence at last follow-up. The preoperative clinical, electrophysiological, and neuroimaging characteristics were explored. More patients with complete resection of the epileptogenic area were likely to obtain seizure freedom outcomes (94.9 *versus* 74.1%, *p* = 0.040). The patients with a duration of epilepsy less than 12 years had increased likelihood of seizure freedom after surgery (69.2 *versus* 44.4%, *p* = 0.044). The patients with ATLR were more likely to obtain seizure freedom (41.0 *versus* 29.6%) but did not reach significance (*p* = 0.344). Moreover, the presence of gray–white blurring or HS on MRI was also more likely to obtain seizure freedom but failed to reach significance (*p* = 0.406 and *p* = 0.539, respectively).

**FIGURE 4:**
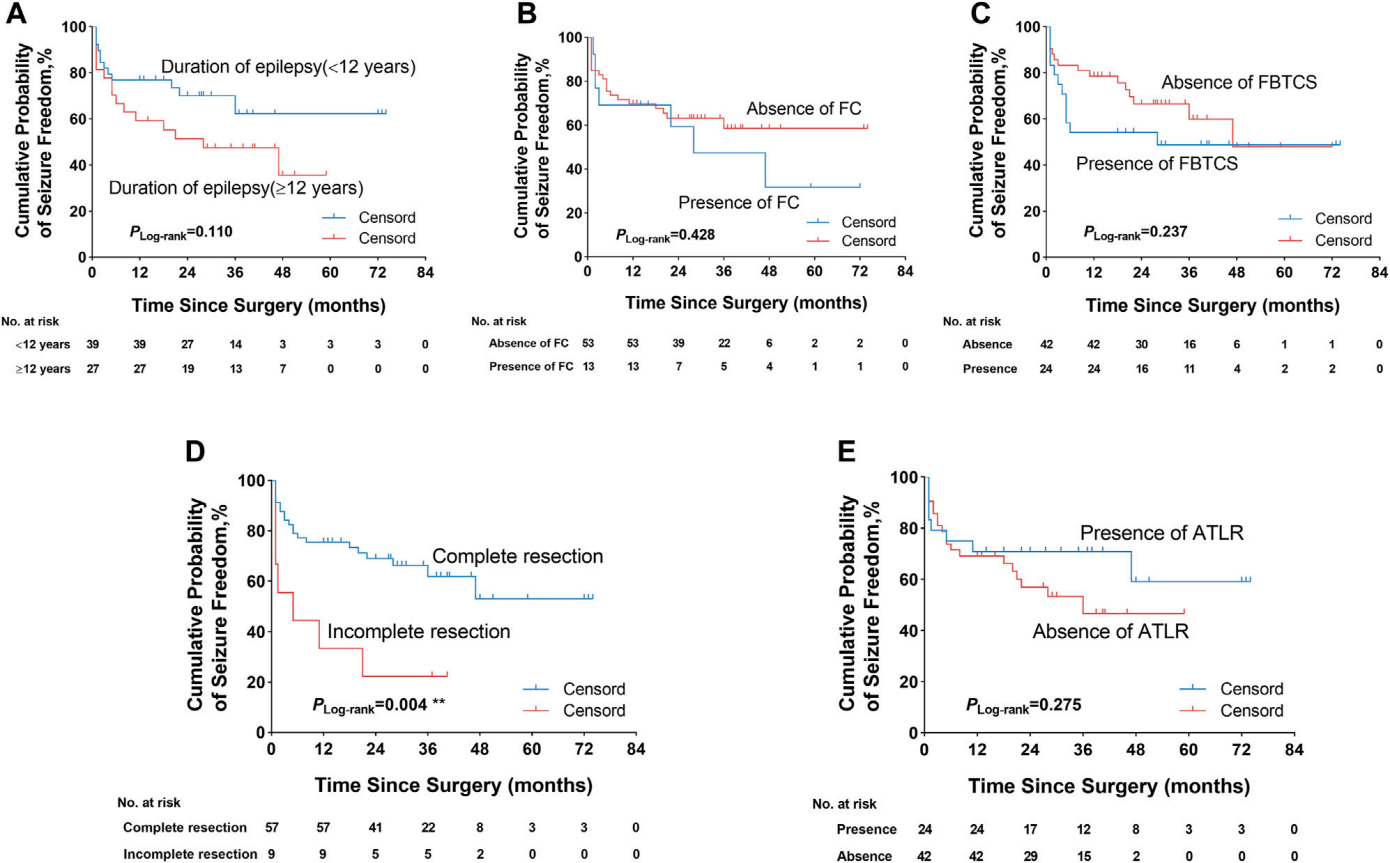
Kaplan–Meier survival curves of Engel class Ia seizure freedom depending on different outcome predictors. **(A)** Duration of epilepsy was not significantly associated with seizure freedom. **(B)** Absence or presence of FC did not significantly influence seizure outcome. **(C)** Absence or presence of FBTCS did not impact seizure outcome. **(D)** Complete resection of the epileptogenic area was significantly more likely to obtain seizure freedom. **(E)** ATLR was also not significantly associated with seizure outcome. **p < 0.01. FC, febrile convulsion; FBTCS, focal to bilateral tonic–clonic seizure; ATLR, anterior temporal lobe resection.

**FIGURE 5:**
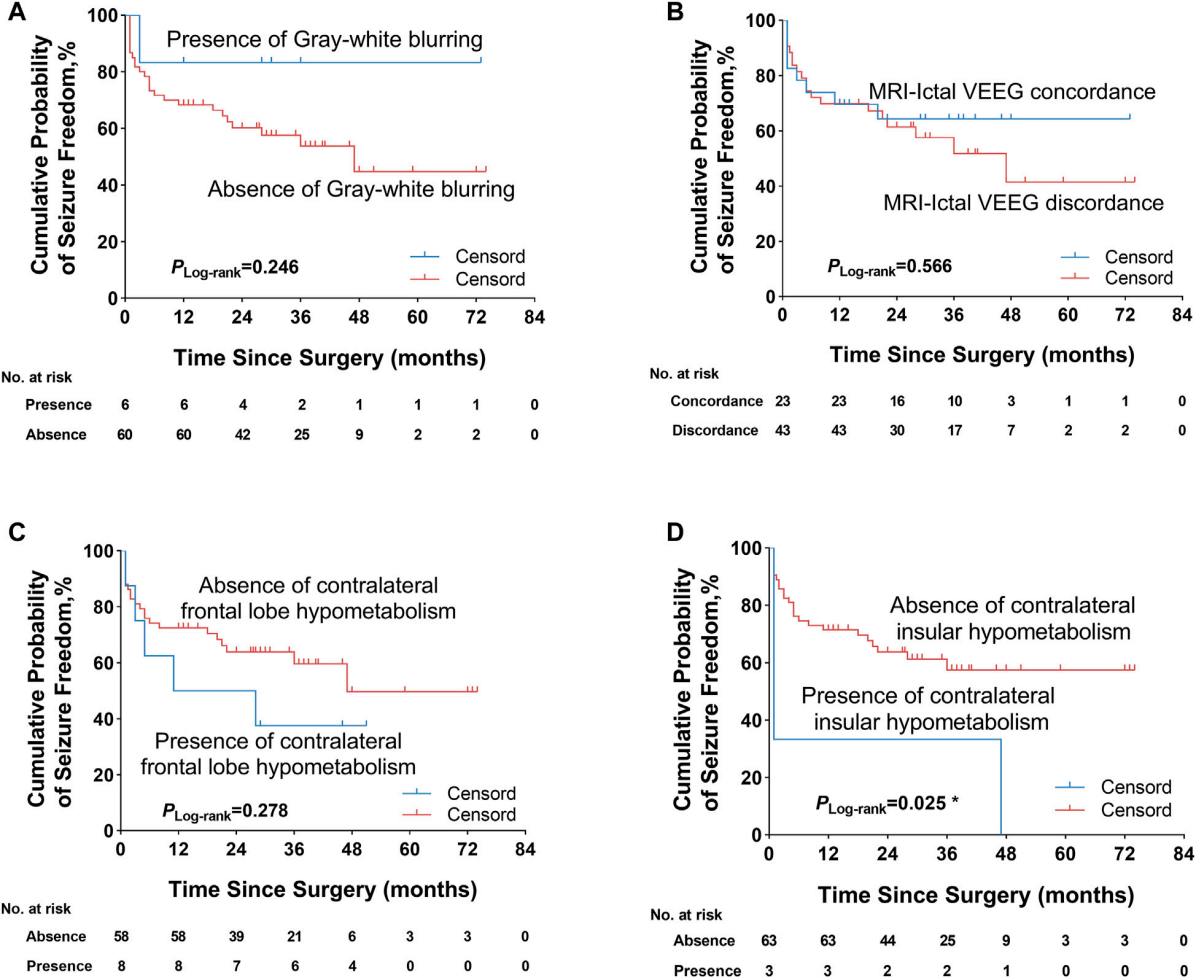
Kaplan–Meier survival curves of Engel class Ia seizure freedom since surgery. **(A)** Absence or presence of gray–white blurring on MRI was not significantly associated with seizure outcome. **(B)** Concordance or discordance of preoperative MRI with ictal scalp VEEG did not significantly influence seizure outcome. **(C)** Absence or presence of contralateral frontal lobe hypometabolism on PET/MRI was also not significantly correlated with seizure outcome. **(D)** Patients without contralateral insular lobe hypometabolism on PET/MRI were significantly more likely to obtain seizure freedom. *p < 0.05. VEEG, video electroencephalogram.

We further explored some univariate predictors utilizing the KM survival curves of seizure freedom outcome (Figures 4, 5). In our included patients, the influence of the two preoperative factors on seizure outcome was statistically significant, as follows (Figure 4D, Figure 5D **)**: 1) the patients with complete resection of the epileptogenic area were significantly more likely to achieve seizure freedom (Engel class Ia) compared with patients with incomplete resection of epileptogenic area (*p*_Log-rank_ = 0.004). 2) Patients without contralateral insular lobe hypometabolism were significantly more likely to achieve seizure freedom as compared with patients with contralateral insular lobe hypometabolism (*p*_Log-rank_ = 0.025). We found that duration of epilepsy (more or less than 12 years), absence or presence of FC or FBTCS, and ATLR did not influence the Engel class Ia outcome (*p*_Log-rank_ = 0.110, *p*_Log-rank_ = 0.428, *p*_Log-rank_ = 0.237, *p*_Log-rank_ = 0.275, respectively) (Figures 4A–C,E). The KM survival curves demonstrated no significant effect of absence or presence of gray–white blurring on MRI on seizure freedom outcome (*p*_Log-rank_ = 0.246) (Figure 5A). There was no significance on Engel class Ia outcome of concordance or discordance of preoperative MRI with preoperative ictal scalp VEEG (*p*_Log-rank_ = 0.566) (Figure 5B). Moreover, the absence or presence of contralateral frontal lobe hypometabolism on PET/MRI did not impact seizure outcome (*p*_Log-rank_ = 0.278) (Figure 5C).

To further confirm the above univariable predictor identified as potentially significant in the KM survival curves analysis, we conducted a multivariable analysis utilizing a Cox proportional hazards regression model (Table 3). In the overall patients, the presence of incomplete resection of the epileptogenic area was the independent significant predictor of seizure recurrence and was correlated with an approximately three-fold increase in the risk of postoperative seizure recurrence (HR = 2.977, 95% CI 1.218–7.277, *p* = 0.017) as compared with patients who underwent complete resection. The predictive value of the presence of contralateral insular lobe hypometabolism on PET/MRI was also investigated, but the predictor failed to reach statistical significance (HR = 2.885, 95% CI 0.819–10.857, *p* = 0.099).

**TABLE 3:** **|** Predictors of postoperative seizure recurrence in FCD type IIIa patients.

Variable	Hazard ratio (HR)	95% CI	*p*
Incomplete resection of epileptogenic area	2.977	1.218–7.277	0.017*
Presence of contralateral insular lobe hypometabolism	2.885	0.819–10.857	0.099

Note. HR, hazard ratio.

*Statistically significant.

## Discussion

Although some studies have investigated the electroclinical, imaging characteristics and predictive factors of postoperative outcomes in the patients with FCD, the features and predictive role for FCD type IIIa patients have yet to be evaluated. This is the first cohort including children and adults followed up 22.0–40.6 months after operative treatment of drug-resistant epilepsy caused by FCD type IIIa according to the new ILAE classification method. In this retrospective study, we demonstrated that duration of epilepsy (less than 12 years), absence of contralateral insular lobe hypometabolism on PET/MRI, and complete resection of the epileptogenic area were associated with seizure outcome by univariate analysis. Multivariate Cox regression analysis indicated that the incomplete resection of the epileptogenic area was the only independent predictor for seizure recurrence after surgery.

In the present study, we showed that FIAS was the most common seizure type in FCD type IIIa patients, followed by FBTCS. The most common symptoms were automatisms and autonomic behavior. A large number of patients (60.6%) with FCD type IIIa had auras. This result was less than 90% reported by Fauser et al. (2013). Moreover, we demonstrated that the patients with ATLR were more likely to obtain seizure freedom but did not reach significance, which was in accordance with the previous finding, which reported that patients with FCD type IIIa were more likely to achieve better seizure outcomes after ATLR compared with HS alone (Duhrsen et al., 2018). We also found that only a fraction of patients with FCD type IIIa had the manifestation of FCD on MRI including gray–white blurring, cortical thickening, and subcortical T2/FLAIR abnormality, which was concordant with the previous findings (D. W. Kim et al., 2012). Paradoxically, Fauser et al. reported that 88% of patients with FCD type IIIa presented with the gray–white blurring or subcortical signal intensity abnormality on MRI. This result also demonstrated that the presurgical identification of FCD type IIIa by MRI remained difficult and doubtful to a certain degree. These comprehensively clinical, electrophysiological and neuroimaging features could sufficiently help the clinician to diagnose and evaluate the patients with FCD type IIIa.

We reported that 59.1% of patients had the postoperative seizure freedom outcome at the last follow-up. This result aligned with the seizure freedom outcome reported by the previous findings (Fauser et al., 2013; Deleo et al., 2016) and worse than the favorable outcome reported by Fauser et al. (2015)and Jayalakshmi et al. (2019). We further investigated that the predictive factors of postoperative seizure freedom were complete resection of the EZ, absence of contralateral insular lobe hypometabolism on PET/MRI, and duration of epilepsy less than 12 years. However, we could not replicate previously reported predictors such as younger age at the surgery, absence of acute postoperative seizure, and intracranial electrode implantation (Kim et al., 2011; Jin et al., 2016; Andrews et al., 2019; Chen et al., 2019).

Our study first indicated that contralateral insular lobe hypometabolism on PET/MRI coregistration was correlated with seizure recurrence in patients with FCD type IIIa but was not an independent predictive factor for seizure outcome by multivariate Cox regression analysis. This was similar to the previous finding reported by Hur et al. (2013), who observed the trend towards a worse postoperative outcome in patients with insular hypometabolism, but the results did not reach significance. It was a sign that the insular cortex present dense interconnection neural network and allowed for the fast spread of ictal epileptic discharges. Such extensive involvement of the ictal discharges led to wider regions of hypometabolism in interictal PET/MRI imaging. Further studies are needed to explore the relationship between the hypometabolism pattern and postoperative outcome.

In accordance with the previous studies (D. W. Kim et al., 2009; Fauser et al., 2015; Oluigbo et al., 2015; Jin et al., 2016), our current study also demonstrated that complete resection of the EZ was the independent predictor of postoperative seizure outcome for FCD type IIIa. This study found that the patients with incomplete resection of the epileptogenic area had an approximately three-fold increase in the risk of seizure recurrence after surgery as compared with those with complete resection. However, our results also indicated that among the patients undergoing complete resection, 35.0% with complete resection of the epileptogenic area had seizure recurrence, which was higher than the previous finding (Jin et al., 2016). This phenomenon possibly resulted from the preoperative inaccurate recognition of the epileptogenic area and the development or maturation of a new EZ (Najm et al., 2013). Moreover, we indicated that the shorter duration of epilepsy (less than 12 years) was also a predictive factor of seizure freedom outcome after surgery, which was in accordance with some studies that duration of epilepsy was significantly associated with surgical outcome (Kral et al., 2003; Fauser et al., 2008; Fauser et al., 2015). This result highlighted that we should consider early epilepsy surgery in patients in whom drug-resistant epilepsy becomes obvious. However, in another study, Jin et al. (2016) did not observe the significant association of duration of epilepsy with surgical outcome.

In the current study, we showed that age at operation and seizure onset was not associated with the seizure outcome, which was in concordance with the previous studies that there was no significant influence of age at operation and seizure onset on postoperative seizure outcome (D. W. Kim et al., 2009; Krsek et al., 2009; Rowland et al., 2012; Jin et al., 2016). However, a large study about FCD including FCD type IIIa demonstrated that a younger age at operation could aid in obtaining seizure freedom (Fauser et al., 2015). In addition, Kim et al. (2011) also reported that younger age at the time of surgery was more likely to achieve good postoperative outcomes. This discordance may have resulted from the various criteria of patient selection and the different FCD subtypes. The relationship between the operation time and postoperative outcome for FCD, especially FCD type IIIa, was needed for further investigation.

Several limitations should be considered in the explanation of these results. Firstly, this is a retrospective study, so the selection bias is inherent to the study. We try to minimize bias as much as possible by performing blinded electrophysiological, imaging, and histopathology review utilizing novel classification methods. Secondly, this single-center and retrospective study limits its general relevance. Moreover, another limitation of our study is that we do not record EEG during the ^18^F-FDG PET scan and cannot exclude ictal scans.

In summary, postoperative seizure outcome in the surgical treatment of FCD type IIIa was in more than half of the patients. This underlines that surgery can be a critically effective therapy option for FCD type IIIa patients. More specifically, we comprehensively concluded the clinical, electrophysiological, and neuroimaging characteristics of FCD type IIIa, and we further investigated the prognostic factors of FCD type IIIa by surgical treatment. The surgical outcome of FCD type IIIa is affected by critical determinants, including duration of epilepsy, complete resection of the epileptogenic area, and contralateral insular lobe hypometabolism on PET/MRI. The complete resection of the epileptogenic area is the only independent predictor for seizure freedom outcome after surgery. With the advancement of both noninvasive multimodality imaging and comprehensive invasive assessment with SEEG, we will further improve preoperative evaluation and the postoperative outcome for patients with intractable epilepsy associated with FCD type IIIa.

## Data Availability

The raw data supporting the conclusion of this article will be made available by the authors, without undue reservation.
